# Supplementary material for: Isolation, Propagation and Genotyping of Human Rotaviruses Circulating among Children with Gastroenteritis in Two Egyptian University Hospitals
Source: Biology (Basel). 2022 Sep 28;11(10):1413. doi: 10.3390/biology11101413 (PMC9598786; doi:10.3390/biology11101413)
Supplement: Supplementary file 1 [file biology-11-01413-s001.zip › biology-1901899-supplementary.pdf]

Table S1. PCR conditions for detection of VP6- rotavirus fragment

Steps	cDNA	1st Denaturation	2nd Denaturation	Annealing	Elongation	Termination	Hold
	One step RT-PCR reaction						
Temperature °C	45	94	94	55	72	72	4
Time	30 min	5 min	30 sec	45 sec	60 sec	5 min	∞
No. of Cycles	1	1	35			1	

Table S2. The primer set used for VP7 fragment G-genotyping

Gene	Primer	Primer sequence (5′ → 3′)	G-Genotype	References
First PCR				
VP7 (*Segment 9*)	Beg9	GGCTTTAAAAGAGAGAATTTCCGTCTGG		[32]
	End9	GGTACACATCATACAATTCTAATCTAAG		
Second PCR				
	End9	GGTACACATCATACAATTCTAATCTAAG		
	aAT8-69M	GTCACACCATTTGTAAATTCG	G8	
	aBT1-Wa	CAAGTACTCAAATCAATGATGG	G1	
	aCT2-DS-1	CAATGATATTAACACATTTTCTGTG	G2	
	aDT4-ST-3	CGTTTCTGGTGAGGAGTTG	G4	[32]
	aET3-P	CGTTTGAAGAAGTTGCAACAG	G3	
	aFT9W161	CTAGATGTAACTACAACTAC	G9	
	G10	ATGTCAGACTACARATACTGG	G10	
	G12	CCGATGGACGTAACGTTGTA	G12	

Table S3. The primer set used for VP4 (P) genotyping

Gene	Primer	Primer sequence (5′ → 3′)	P-Genotype	References
First PCR				
VP4 (*Segment 4*)	Con2	ATTTCGGACCATTTATAACC		[33]
	Con3	TGGCTTCGCTCATTTATAGACA		
Second PCR				
	Con3	TGGCTTCGCTCATTTATAGACA		
	1-TI-KU	ACTTGGATAACGTGC	P[8]	
	2T-1	CTATTGTTAGAGGTTAGAGTC	P[4]	
	3T-1	TGTTGATTAGTTGGATTCAA	P[6]	[33]
	4T-1	TGAGACATGCAATTGGAC	P[9]	
	5T-1	ATCATAGTTAGTAGTCGG	P[10]	

Table S4. Interpretation of human rotavirus genotyping [32,33,34]

G-types	Amplicon size (bp)	P-types	Amplicon size (bp)
Beg9/End9	1062	Con2/Con3	868
G1	749	P[4]	483
G2	625	P[6]	267
G3	374	P[8]	345
G4	583	P[9]	391
G8	885	P[10]	594
G9	306		
G10	397		
G12	512		

Appendix

A1. Molecular typing of positive samples using VP7 and VP4 segments experimental details.

In the first run, 5 µl of previously isolated RNA was combined with 1 µl of each VP7 specific primer pairs (Beg9/End9). To denature the dsRNA, the mixture was heated at 70°C for 5 minutes before being chilled in an ice bath for 2 minutes. The RT-PCR reactions were then carried out in two phases. In the first phase, reverse transcription was done on denatured dsRNA using the cDNA Synthesis Kit for RT-PCR (Catalog number: 30110750) (iNtRON Biotechnology, Korea). The cDNA yield was amplified in the second phase using FastStartTM PCR Master (Merck, Germany). Agarose gel electrophoresis (1.5%) was used to separate the amplicons. A second PCR run was performed to determine the genotype of the isolated rotavirus strains using the reverse primer and a cocktail of primers. In brief, 1 μl of the first-round amplicons were mixed with 40 μl of PCR-Master and 1 μl of each of the End9 and G genotype-specific primers (Supplementary Table S2). In the second run, the same PCR conditions as previously mentioned were utilized. The amplicons were separated using 1.5% agarose gel electrophoresis, and the genotype was determined based on the product size [34]. Same techniques and settings, that were used to detect G genotypes, were used to detect P genotypes, but this time a VP4 specific primer pairs (Con2/ Con3) was used as terminal primers in the first amplification round and Con3 as well as a cocktail of P genotype-specific primers in the second one (Supplementary Table S3).
